# Supplementary material for: Feature representation in analysing childhood vaccination defaulter risk predictors: A scoping review of studies in low-resource settings
Source: PLOS Digit Health. 2025 Jul 30;4(7):e0000965. doi: 10.1371/journal.pdig.0000965 (PMC12310020; doi:10.1371/journal.pdig.0000965)
Supplement: S1 Appendix — The supplementary narratives contain summaries for each of the studies reviewed: the authors, predictors identified in the articles, the methods used to predict the outcome of interest, and the outcomes of interest predicted. (DOCX) [file pdig.0000965.s003.docx]

**S1 Appendix**

Nantongo and colleagues [1] pioneered machine learning to predict routine immunization defaulting in Uganda, leveraging District Health Surveillance (DHS) data from 2016. By using Principal Component Analysis for dimensionality reduction, they employed models such as k-Nearest Neighbours, Decision Trees, Random Forests, Support Vector Machines, Naïve Bayes, Logistic Regression, XGBoost, AdaBoost, and Gradient Boosting. The predictors varied by vaccine and included socio-economic, maternal, child, and community-level factors. Among the most influential features were immunization history, parental characteristics, and location-based factors. Despite its robust methodology, the study faced limitations due to an older dataset and class imbalance issues.

Abateman and colleagues [2] used primary data to analyse predictors of full childhood vaccination status in Ethiopia. The study employed logistic regression methods to identify significant factors, including maternal education level, type of community settlement, mothers' knowledge of childhood vaccination benefits, and reminders for the next vaccination date.

Analysing data from 441 children using binary logistic regression, Abegaz and colleagues [3] found multiple predictors influencing vaccination coverage in Ethiopia. Among the most significant were maternal age, delivery location, knowledge of immunization benefits, travel time, marital status, maternal education, antenatal care (ANC) visits, and reminders. The study underscored the role of ANC visits and maternal education in improving vaccination rates.

Using logistic regression on secondary data from 1,522 children, Muhoza and colleagues [4] examined the role of maternal age, reminders, settlement type, birth order, and place of residence in determining the uptake of the second-year life dose of measles and Meningococcal Serogroup A vaccinations in Ghana. The analysis suggested that urban children and those with younger mothers had higher immunization rates, emphasizing the importance of tailored reminder systems.

With a dataset of approximately 95,000 children from India, Mali, and Nigeria, Biswas and colleagues [5] leveraged machine learning techniques, including cost-sensitive Ridge Classification, Nearest Neighbour, and Multilayer Perceptron. The findings revealed that place of residence, delivery location, and ANC visits were crucial factors, with urban areas and health facility births serving as strong indicators of better vaccination outcomes.

The study by Demash and colleagues [6] assessed childhood vaccination completeness using data from 1,617 Ethiopian children. Machine learning models, including Naïve Bayes, PART, logistic regression, multilayer perceptron, J48, Logit Boost, random forest, and AdaBoost, were applied after preprocessing and balancing the dataset. PART emerged as the best-performing model with an accuracy of 95.53%. The top predictors were ANC visits, institutional delivery, health facility visits, maternal education, and wealth status. The study limited predictors to socio-demographic characteristics.

Drawing from DHS data on approximately 21,000 children in Nigeria, Aheto and colleagues [7] employed logistic regression to assess vaccination predictors on the receipt of three different vaccines. Key determinants included vaccination card availability, maternal age, vitamin A supplementation, maternal employment, education, religious affiliation, phone/internet access, ethnicity, ownership of a bank account, livestock ownership, and travel time. The study highlighted the multifaceted socio-economic barriers to immunization.

Santos and colleagues [8] conducted a Classification and Regression Tree analysis of DHS and Investigator-generated data (IGD) covering over 210,000 children in 92 low-and-middle income countries to identify predictors of children with high risk of zero-dose vaccination. Significant predictors included ANC visits, place of delivery, maternal tetanus vaccination status, wealth index, settlement type, and maternal education. A particularly vulnerable group was identified based on the combination of no ANC visit, home delivery, and lack of maternal tetanus vaccination. The notion of a composite predictor was seen in this study as a triple predictor feature having higher significance than the individual predictors.

Touré and colleagues [9] investigated full childhood vaccination determinants among 380 children in Guinea using logistic regression. The findings pointed to vaccination card availability, ANC visits, birth order, child’s sex, place of delivery, residence, illness before the scheduled vaccination date, and awareness of vaccination benefits as key influences on immunization rates.

Investigating the non-utilization of measles vaccination in Bangladesh, Hasan and colleagues [10] analysed DHS data from 2007–2018 using Synthetic Minority Over-sampling Technique (SMOTE) to handle class imbalance. A range of classifiers, including Gaussian and Bernoulli Naïve Bayes, Decision Tree, Random Forest, XGBoost, LightGBM, and an ensemble model, were tested. Performance was evaluated through sensitivity, precision, accuracy, and ROC-AUC, with statistical significance confirmed via ANOVA and T-tests. Key predictors included birth order, caregiver’s age, ANC visits, household size, and media exposure. While robust, the study could benefit from more recent data.

Employing multivariate statistical analysis and binary logistic regression, Budu and colleagues [11] analysed DHS data from 5,119 children in Ghana for full dose reception. The results indicated that maternal education, religious affiliation, settlement type, ethnicity, parity, wealth index, and place of residence significantly impacted vaccination uptake, with higher socio-economic status correlating with improved immunization coverage.

The Somalia study by Jama [12] involved a descriptive statistical approach to analyse determinants of complete vaccination among 315 children aged 11–24 months and found that maternal education, place of delivery, and travel time were crucial factors. Institutional deliveries and shorter travel distances were linked to increased immunization rates.

Mohanraj G et al. [13] focused on predicting vaccination rates in low-immunization regions of India using a hybrid deep-learning framework. DHS data from 5,057 children were analysed using Rank-Based Multi-Layer Perceptron (R-MLP), Deep Soft Cosine Semantic models, Ranking SVM, and traditional classifiers like Decision Trees, Naïve Bayes, and Linear Regression. The study utilized 28 predictors, emphasizing socio-demographic factors, ANC visits, and vaccination history. Performance was evaluated through precision, recall, accuracy, and the F1-measure, but details on regional variations were not extensively explored.

Acharya and colleagues [14] used logistic regression on 4,330 records from DHS data in Nepal to identify predictors of vaccination rates, whiles closely examining inequalities by wealth quintile and maternal education. The authors identified settlement type, maternal education levels and sex of child as significant predictors of full immunization. The also laid emphasis on the persistently higher impact of maternal education inequalities on vaccination rates compared to wealth inequalities.

Adamu and colleagues [15] applied a combination of logistic regression and Markov Chain Monte Carlo methods to analyse data from 675 children attending health facilities in Nigeria. The objective was to identify predictors of missed opportunities for routine childhood vaccination. The study identified travel time, the number of vaccinators, birth order, child’s age, type of health facility, and caregiver’s demographic characteristics as significant predictors of at least one missed opportunity for routine childhood vaccination.

Acharya and colleagues [16] provided interesting insights into the complex interplay of individual and community-level predictors on full childhood vaccination in the Democratic Republic of Congo (DRC) by using DHS data on 3,366 children and logistic regression. Significant individual-level factors identified included sex of child, delivery location, parental demographics, ANC and postnatal care (PNC) visits, economic autonomy, wealth, travel time. On the other hand, significant community-level factors spanned poverty rates, maternal unemployment rates, maternal education rates, and facility delivery rates among others.

Applying binary logistic regression and econometric methods to DHS data from 6,533 children in Ghana, Asuman and colleagues [17] highlighted key predictors full childhood vaccination such as maternal age, education, employment, marital status, place of delivery, religious affiliation, health insurance coverage, and wealth index. Socioeconomic disparities played a substantial role in immunization rates.

Sheikh and colleagues [18] used logistic regression on DHS data from 1,631 children to analyse the determinants of timelines and completeness of immunization in Bangladesh. The study revealed that seasonal birth patterns, maternal employment, household size, education, travel time, wealth index, maternal age, and sanitation quality were all significant in determining vaccination uptake. The study provided insights for considering climate effects on the completeness and timeliness of immunization.
