# Supplementary material for: Feature representation in analysing childhood vaccination defaulter risk predictors: A scoping review of studies in low-resource settings
Source: PLOS Digit Health. 2025 Jul 30;4(7):e0000965. doi: 10.1371/journal.pdig.0000965 (PMC12310020; doi:10.1371/journal.pdig.0000965)
Supplement: Attachment — Submitted filename: Response to Reviewer comments.docx [file pdig.0000965.s005.docx]

We would like to express our sincere gratitude to our reviewers for the insightful comments. Addressing these comments have helped us further improve the quality of our manuscript. Kindly see below the responses on how each comment received have been addressed in the manuscript.

**Response to Editor comments**

**Comment:** The authors address a highly relevant and timely issue in which artificial intelligence plays a crucial role: the representation, encoding, and engineering of predictors used to assess the risk of childhood vaccination default in low-resource settings. However, the manuscript, in its current form, is not suitable for publication in PLOS Digital Health. There are several structural and methodological aspects that need improvement to enhance the clarity, reproducibility, and overall impact of the work. These include data availability, the selection and definition of predictor variables, modelling approaches, feature encoding strategies, and the inclusion of a systematic comparison with prior studies—preferably summarized in a table highlighting the differences. Additionally, the manuscript should report the results of both statistical and machine learning methods more clearly. Finally, issues related to grammar and punctuation must also be corrected.

**Response**:

Data availability: We have developed a structured data extraction file capturing key study-level variables: country context, study design, predictors analysed, encoding strategies used, analytical methods applied, and outcome definitions. This dataset is available as a supplementary Excel file.

selection and definition of predictor variables: We have introduced Table 2 that describes the top ten predictor variables identified in the studies reviewed as well as their frequency of occurrence. In addition to this, a separate sub-section titled “Definition and Classification of Features” has been introduced to bring more clarity to the section that describes the predictor variables. We also moved Table 5, which summarizes definitions of predictor variables before the sub-section on feature encoding, engineering and representation to improve the flow and clarity of the manuscript.

modelling approaches: Table 3 has been included to provide a summary of the modelling methods used across the reviewed studies and the studies that leveraged the models.

feature encoding strategies: Results on the encoding strategies and frequency of occurrence across the studies have been included and presented in Table 4.

inclusion of a systematic comparison with prior studies: We have included in the discussion section Table 8, which contrasts the current review with prior works based on key aspects.

manuscript should report the results of both statistical and machine learning methods more clearly: We have created a result sub-section titled “Analytical Summary of Reviewed Studies”. In this section, we have presented descriptive and analytical summaries (Table 2, 3 and 4) derived from the reviewed studies. Summaries show distribution of top 10 predictors across studies, distribution of modelling methods used across studies, and the encoding strategies used by various studies. We have improved the general workflow of the results section and included a table (Table 7) that focuses on the reported performance results of models from machine learning-based studies included in this scoping review.

**Response to Reviewer 1 comments**

**Comment 1**: The manuscript lacks transparency in terms of data availability. Although the authors state that data were extracted from publicly available studies, they do not include the full data extraction sheet or a structured summary of the included studies in a machine-readable format (e.g., .csv or .xlsx). For a scoping review, this level of transparency is essential. A detailed supplementary file outlining study characteristic, predictor variables, modelling approaches, and feature encoding strategies should be made available to ensure reproducibility and enable other researchers to build upon the findings

**Response 1**: To enhance transparency and reproducibility, we have developed a structured data extraction file capturing key study-level variables: country context, study design, predictors analysed, encoding strategies used, analytical methods applied, and outcome definitions. This dataset is available as a supplementary Excel file. We have also revised the methods sub-section on “Data Charting and Analysis” to mention the inclusion of this file (lines 183 – 186). We have also revised the data availability statement to reflect the inclusion of this file.

**Comment 2**: While the manuscript presents a narrative synthesis of the included studies, it lacks a systematic comparison across them. The addition of a clear summary table mapping predictors to encoding strategies, model types, and outcome definitions would provide much-needed structure and allow for clearer interpretation of patterns and gaps in the literature. Without this, readers are left with descriptive lists that are difficult to synthesize or act upon

**Response 2**: In response to the need for a more systematic comparison across studies, we developed a summary table (Table 6) mapping key predictors to their corresponding encoding strategies, model types, and outcome definitions. The table allows for structured synthesis and easier interpretation of how different features have been operationalized in the context of childhood vaccination defaulter risk modelling. It reveals patterns in the use of binary encoding for categorical data, common reliance on logistic regression or ensemble models, and a shared focus on complete or timely vaccination as the primary outcome. We also moved (Table 5) that summarized predictor definitions before the sub-section on feature encoding, engineering and representation to improve the flow of the manuscript.

**Comment 3**: The manuscript states that both statistical and machine learning methods were used for analysis, but the results of these analyses are not presented in the Results section or in the appendices. Even partial findings—such as frequency counts, clustering summaries, or principal components used—would help validate the claims made and enhance the rigor of the review. The absence of this information undermines the strength of the conclusions and leaves the reader uncertain about the analytical contributions of the study

**Response 3**: To address this reviewer comment, we have created a sub-section titled “Analytical Summary of Reviewed Studies” which starts from page 15 (line 217). In this section, we have presented descriptive and analytical summaries (Table 2, 3 and 4) derived from the reviewed studies. Summaries show distribution of top 10 predictors across studies, distribution of modelling methods used across studies, and the encoding strategies leveraged by various studies. We further introduced a sub-section titled “Classification of Features” (Page 17, line 255) to separate the Analytical summary from the subsequent section that describes the features in broad categories.

**Response to Reviewer 2 comments**

**Comment 1&2**: I suggest reviewing the manuscript for punctuation and grammar errors, particularly in the Results section. In the Methods section, “Search Strategy” paragraph, line 153, I advise specifying the end month (i.e., January 2018) for consistency with the reported end date

**Response 1&2**: Punctuations and grammar errors have been reviewed and revised appropriately as suggested. The revision has been made throughout the manuscript as suggested

**Comment 3**: There are several instances throughout the manuscript where in-text citations did not mention author names and only included numbers

**Response 3**: The names of the authors have been included at the appropriate in-text citation points as suggested

**Comment 4**: In the PRISMA chart (Figure 1), it is not clear that the total number of included articles is 18, as noted in Table 1, line 191, and line 198. Further clarification is needed to distinguish between “New studies included in review n=4” and “Reports of new included studies n=4”. I suggest reviewing the PRISMA chart to ensure consistency with the text.

**Response 4**: A new PRISMA chart has been generated for figure 1 to make the information clearer. The line labelled “Reports of new included studies n=4” has been removed. Also, a correction was made to reflect that all 55 reports were successfully retrieved and so 55 reports were assessed for eligibility instead of 53.

**Comment 5&6**: In Table 1, row 4, column 6, there is missing information regarding the outcome of the study by Biswas, A, et al. In Table 2, punctuation should be reviewed as there are a few instances where periods start before the in-text citation

**Response 5&6**: Table 1 has been updated to provide the missing information related to the study by Biswas, A, et al. Also, we have addressed inconsistencies in punctuations and grammatical errors as suggested.
